# Supplementary material for: Serum sCD40L and IL-31 in Association with Early Phase of IgA Nephropathy
Source: J Clin Med. 2023 Mar 3;12(5):2023. doi: 10.3390/jcm12052023 (PMC10004527; doi:10.3390/jcm12052023)
Supplement: Supplementary file 1 [file jcm-12-02023-s001.zip › jcm-2189186-supplementary.pdf]

Table S1. Cytokine profiles in each group

	IgAN (n = 114)			ADPKD (n = 10)			Controls (n = 5)		
	Detectable samples (%)	Range (pg/mL)	Median detectable (pg/mL)	Detectable samples (%)	Range (pg/mL)	Median detectable (pg/mL)	Detectable samples (%)	Range (pg/mL)	Median detectable (pg/mL)
IL-1β	52 (45.6)	1.95-12	4.41	0	-	0.0	0	-	0.0
IL-4	8 (7.01)	6.05-13.2	7.3	0	-	0.0	0	-	0.0
IL-6	62 (54.3)	5.68-33.5	11.6	0	-	0.0	0	-	0.0
IL-10	2 (1.75)	10.9-30.2	20.5	0	-	0.0	0	-	0.0
IL-17A	1 (0.87)	-	18.8	0	-	0.0	0	-	0.0
IL-17F	5 (4.38)	17.0-47.7	23.6	1 (10)	-	18.6	0	-	0.0
IL-21	1 (0.87)	-	210	0	-	0.0	0	-	0.0
IL-22	2 (1.75)	23.5-261	142	0	-	0.0	0	-	0.0
IL-23	2 (1.75)	151-166	158	0	-	0.0	0	-	0.0
IL-25	1 (0.87)	-	12.1	0	-	0.0	0	-	0.0
IL-31	112 (98.2)	182-543	348	0	-	0.0	0	-	0.0
IL-33	12 (10.5)	25.9-55.3	32.6	0	-	0.0	0	-	0.0
IFN-γ	8 (7.01)	8.63-21.3	14.9	0	-	0.0	0	-	0.0
sCD40L	114 (100)	252-560	367	10 (100)	55.4-146	86.9	0	-	0.0
TNF-α	112 (98.2)	16.0-53.9	27.9	7 (70)	3.64-5.66	4.29	0	-	0.0

Data are expressed as the median (interquartile) or number (percentage). ADPKD, autosomal dominant polycystic kidney disease; IgAN, IgA nephropathy; IFN, interferon; IL, interleukin; sCD40L, soluble CD40 ligand; TNF, tumor necrosis factor.

Table S2. Multivariate-adjusted regression analysis of predictors of serum sCD40L in the cohort of IgAN and ADPKD patients and healthy controls.

	β	P	model R^2
IgAN (vs. not IgAN)	165	<0.0001*	0.24

Adjusted for age and eGFR. *p<0.05.

Multivariate-adjusted regression analysis of predictors of serum IL-31 in the cohort of IgAN and ADPKD patients and healthy controls.

	β	P	model R^2
IgAN (vs. not IgAN)	181	<0.0001*	0.23

Adjusted for age and eGFR.

IgAN, IgA nephropathy; ADPKD, autosomal dominant polycystic kidney disease; eGFR, estimated glomerular filtration rate. *p<0.05.

Table S3. Multivariate-adjusted regression analysis of predictors of eGFR in different models including serum sCD40L in IgAN

	β	P	model R^2
Model 1			
sCD40L ≥ median (pg/mL)	1.68	0.38	0.45
Model 2			
sCD40L ≥ median (pg/mL)	1.12	0.57	0.46
UPCR ≥ 0.5 (g/gCr)	-2.88	0.14	
Model 3			
sCD40L ≥ median (pg/mL)	-0.043	0.98	0.49
UPCR ≥ 0.5 (g/gCr)	-1.86	0.34	
MBP ≥ median (mmHg)	-0.43	0.0073*	

Adjusted for age. The median values of serum sCD40L is 367 (pg/mL).

eGFR, estimated glomerular filtration rate; IgAN, IgA nephropathy; MBP, mean blood pressure; sCD40L, soluble CD40 ligand; UPCR, urinary protein to creatinine ratio. *p<0.05.

Table S4. Multivariate-adjusted regression analysis of predictors of eGFR in different models including serum IL-31 in IgAN

	β	P	model R^2
Model 1			
IL-31 ≥ median (pg/mL)	2.38	0.22	0.44
Model 2			
IL-31 ≥ median (pg/mL)	1.91	0.33	0.45
UPCR ≥ 0.5 (g/gCr)	-2.82	0.15	
Model 3			
IL-31 ≥ median (pg/mL)	1.69	0.39	0.47
UPCR ≥ 0.5 (g/gCr)	-2.56	0.19	
MBP ≥ median (mmHg)	-2.96	0.13	

Adjusted for age. The median values of serum IL-31 is 348 (pg/mL).

eGFR, estimated glomerular filtration rate; IgAN, IgA nephropathy; IL-31, interleukin 31; MBP, mean blood pressure; UPCR, urinary protein to creatinine ratio.

Figure S1. Relationship between Gd-IgA1 and IgAN, proteinuria (UPCR), sCD40L or IL-31 in IgAN patients.

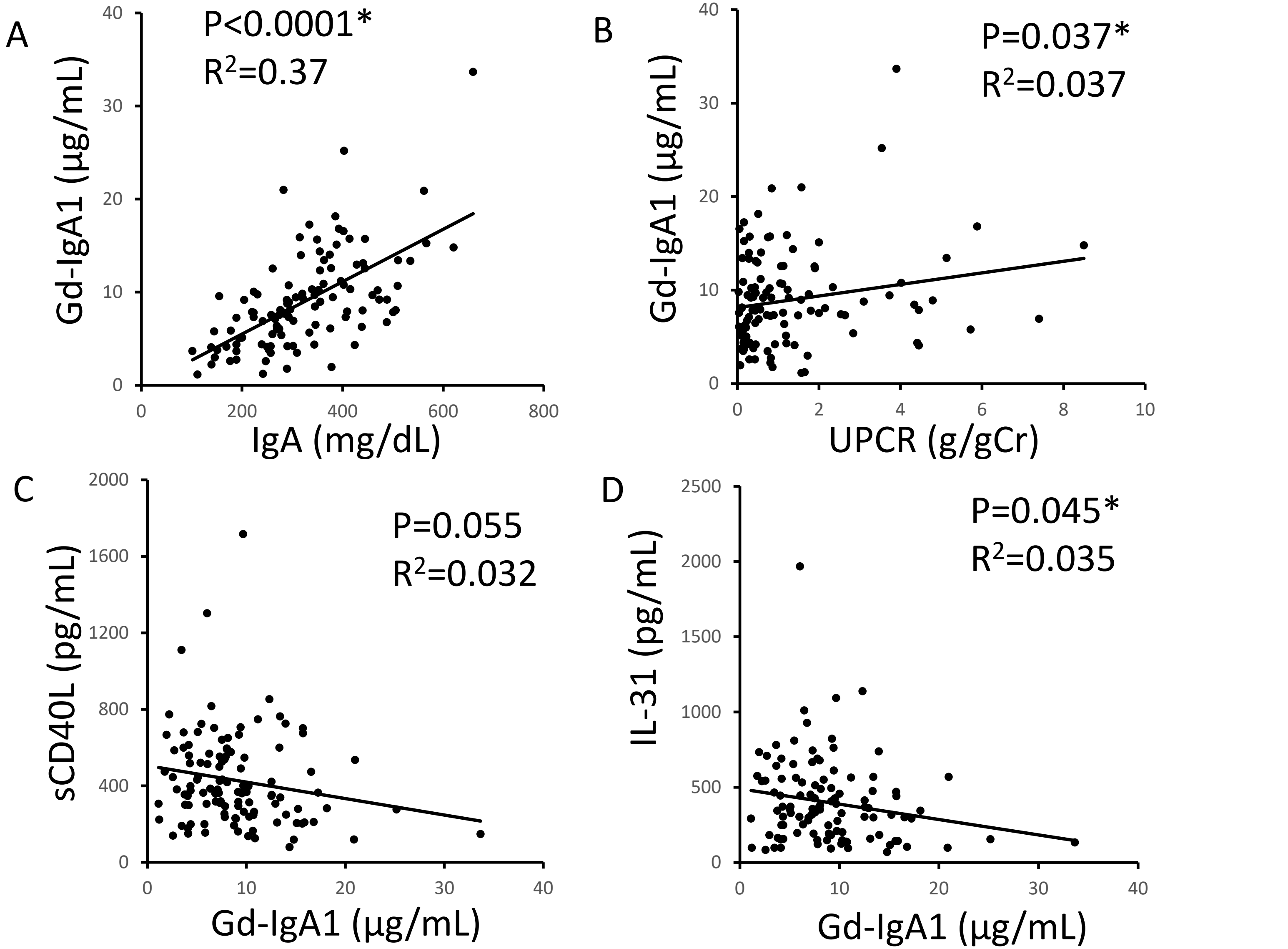

Gd-IgA1 was significantly correlated with serum IgA (A) and the UPCR (B). Gd-IgA1 was not positively correlated with serum sCD40L (C) and IL-31 (D). **p*<0.05.

Figure S2. Relationship between TNF-*α* and proteinuria (UPCR), the renal function (eGFR), sCD40L, IL-31 or Gd-IgA1 in IgAN patients.

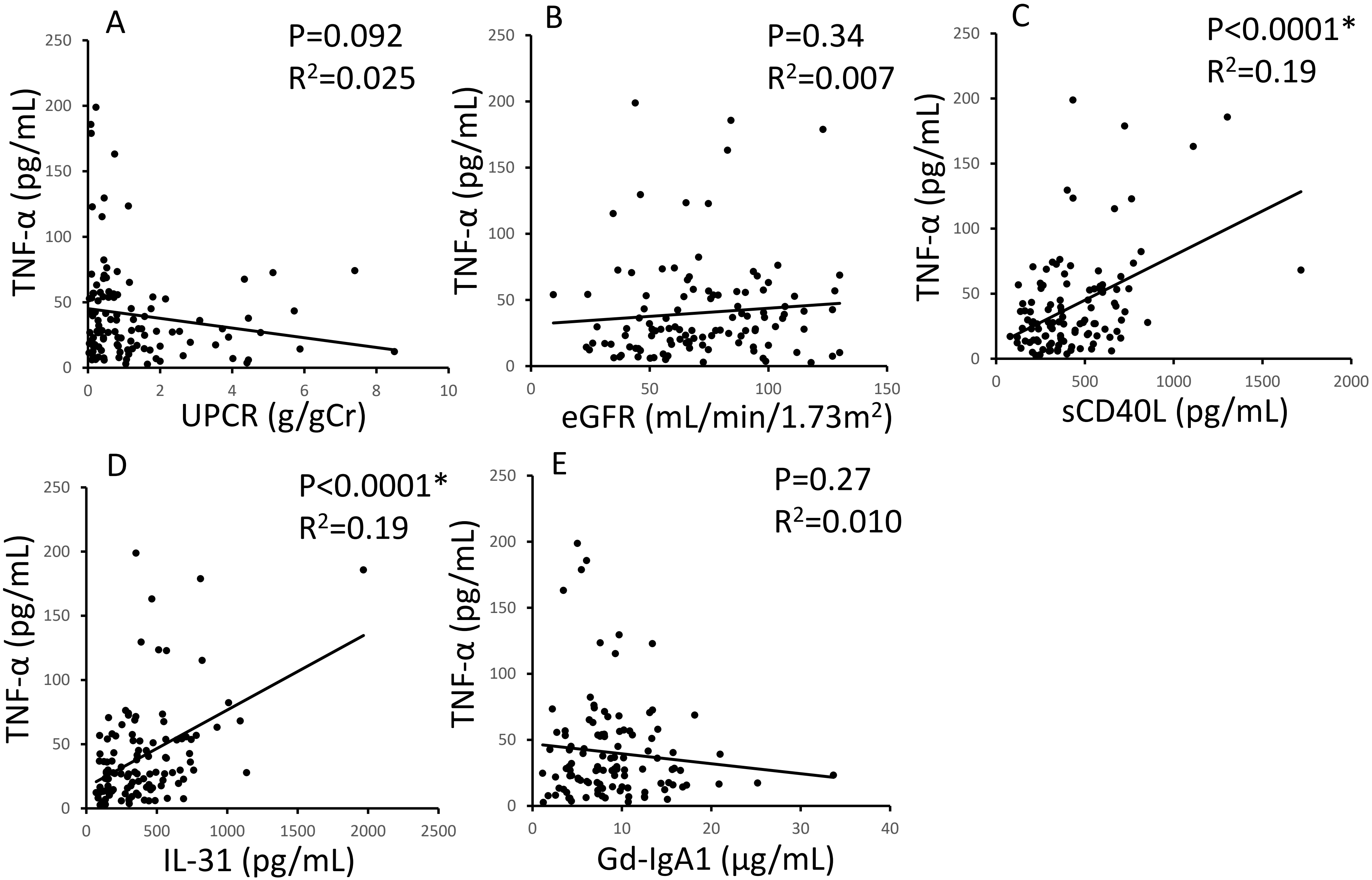

TNF-*α* was not significantly associated with the UPCR (A) or eGFR (B). TNF-*α* was significantly correlated with sCD40L (C) and IL-31 (D), but not with Gd-IgA1 (E). *$p < 0.05$.
